# Patient Counseling Strategies at Female Genital Fistula Repair: A Qualitative Study among Ugandan Fistula Care Providers

**DOI:** 10.21203/rs.3.rs-7331295/v1

**Published:** 2025-09-11

**Authors:** Alison M. El Ayadi, Hadija Nalubwama, Umar Senoga, Maddie Wong, Monica Getahun, Cynthia C. Harper, Suvarna Kantipudi, Abner P. Korn, Justus K. Barageine

**Affiliations:** University of California San Francisco; Makerere University; Makerere University; University of California San Francisco; University of California San Francisco; University of California San Francisco; Sri Ramachandra Institute of Higher Education and Research; University of California San Francisco; Makerere University

**Keywords:** patient-provider communication, health education, surgical recovery, clinical guidelines, female genital fistula, obstetric fistula

## Abstract

**Objective:**

Ensuring high-quality provider counseling is needed to protect the health and well-being of women affected by genital fistula. We sought to understand Ugandan providers counseling practices and strategies.

**Methods:**

We conducted in-depth interviews with 30 fistula providers to understand their counseling practices and recommendations regarding post-repair reproductive health and pregnancy. We analyzed the data thematically across counseling domains: sexual resumption, post-repair pregnancy timeline, and post-repair birth mode.

**Results:**

Providers universally advised delaying sexual intercourse for 3–6 months post-repair to ensure proper healing. While nearly all providers emphasized delaying pregnancy, recommended timelines varied (3 months to >2 years) and were often tailored to patient characteristics and fistula history. Adherence strategies included couples counseling and the provision of written guidance. Contraceptive counseling and provision varied by facility affiliation and fistula type/severity, with religiously affiliated facilities favoring natural family planning. Elective cesarean section for post-repair births was universally recommended and providers employed diverse strategies to overcome the absence of standard guidelines and strong care linkages. Systemic barriers, such as contraceptive stock-outs and fragmented follow-up care, undermined effective counseling. Cross-cutting strategies to improve post-repair outcomes included economic empowerment, male partner engagement, and peer counseling.

**Conclusion:**

Counseling importance and broad messaging was consistent across reproductive health topics; however, key details varied by provider, facility, and patient characteristics. Standardizing key guidance and identifying optimal patient adherence messaging could improve post-repair pregnancy outcomes among this higher-risk population.

## Introduction

Obstetric fistula, a preventable yet debilitating condition, affects a substantial number of women globally, predominantly in sub-Saharan Africa.^1^ Caused primarily by prolonged obstructed labor or surgical error or complication, fistula can result in urinary and/or fecal incontinence, nerve damage, cervical injuries, and pelvic bone trauma, often leading to secondary sequelae.^2–5^ Consequently, affected women face stigmatization and marginalization, hindering their social, economic, and religious participation and contributing to high psychiatric morbidity.^4,6–9^

Evidence suggests that adverse fistula-related impacts may extend into the post-repair period, even where fistula repair has been successful. Women still face adverse fistula-related health outcomes, including poor perinatal outcomes in subsequent pregnancies such as fistula recurrence, spontaneous abortion, and elevated rates of stillbirth.^8,10–12^ Fistula recurrence remains a prevalent maternal complication and is a major source of fear among affected women.^13^ For example, a scoping review of 16 studies revealed an 8% stillbirth rate post-repair overall.^12^ A more recent retrospective cohort study in Uganda identified 14% of first post-repair pregnancies resulted in spontaneous abortion and 5% stillbirth, reflecting a significantly higher stillbirth rate than among a matched external control group.^14^ Both vaginal delivery and emergency cesarean section have been associated with elevated risks of stillbirth and fistula recurrence compared to elective cesarean section.^10,12,14,15^ Elective cesarean sections have been associated with a significant decrease in post-repair stillbirth.^5,6,15^

To mitigate the risk of adverse outcomes, women undergoing fistula repair are encouraged to avoid immediate resumption of sexual activity and delay subsequent pregnancy until a minimum of nine months to one year, ensure early initiation and appropriate use of quality antenatal care services, and plan for an elective (pre-labor) cesarean birth at a capable healthcare facility.^16,17^ Women with fistula are advised to consult a healthcare provider for contraceptive counseling and method access as needed.^17^ Despite current recommendations for elective cesarean section in post-repair births to protect the previously repaired tissue and to improve maternal and neonatal outcomes,^12^ over half of women do not achieve elective cesarean,^10–12,14,18^ highlighting an important disconnect between provider recommendations and post-repair pregnancy-related behaviors and care access.

Recognizing the significant physical, psychosocial, and economic consequences of fistula, and the potential for adverse outcomes in subsequent pregnancies, effective post-repair counseling is increasingly recognized as a crucial component of comprehensive fistula care.^19^ Post-repair counseling should incorporate best practices for optimizing physical and psychosocial recovery which include reproductive and other health education. Studies emphasize informing women about fistula causes and prevention, dispelling stigma, and offering psychosocial support for improved self-esteem and social functioning. Counseling also guides family planning, safe timing for future pregnancies, and the necessity of antenatal care and planned CS delivery.^3–5^ Post-repair counseling presents a potential solution by addressing the consequences of fistula and mitigating the risk of adverse post-repair outcomes.

As the international community aims to move toward a fistula-free generation, protecting the health and well-being of women affected by fistula is crucial. Provider counseling to optimize fistula repair outcomes is critical for women to receive the best possible care and guidance throughout the fistula care continuum. A comprehensive understanding of current post-repair provider counseling practices and strategies is necessary to inform interventions to improve women’s health following fistula repair. This analysis aimed to understand fistula provider counseling practices at the time of repair, including the guidance provided and specific strategies used for effectiveness.

## Methods

As a part of a larger a mixed-methods study in Uganda examining women’s perinatal experiences and outcomes after female genital fistula repair,^15^ we conducted a qualitative sub-study to explore fistula care providers’ perspectives and recommendations for optimizing post-repair outcomes. In Uganda, the proportion of reproductive-aged women reporting fistula-related symptoms is comparatively high.^20,21^ We explored provider perspectives on fistula patients’ (i) post-repair reproductive intentions, (ii) risks associated with post-repair pregnancy, (iii) risk reduction opportunities, (iv) counseling practices regarding post-repair reproductive health and pregnancy (e.g., resumption of sexual activity, family planning, pregnancy, childbirth, and infertility concerns), (v) counseling strategies, (vi) patient adherence, (vii) post-repair pregnancy healthcare needs, and (viii) the adequacy of the current system in meeting those needs. In the current analysis, we review provider counseling practices and strategies across these key content areas.

### Study setting and participants

We recruited 30 fistula care providers from various health facilities across Uganda, including government hospitals, mission hospitals, and not-for-profit hospitals. We purposively selected diverse provider cadres (i.e., fistula surgeons, nurses, midwives, and social workers) and invited them to participate in in-depth interviews via telephone. Most providers were known to study investigators due to their ongoing engagement in fistula clinical care, training, and research in Uganda. The rest were identified through referrals from fistula surgeons.

### Data collection

Trained qualitative researchers (HN and US) scheduled interviews among individuals who were interested in participating and led participants through an informed consent process for which written confirmation was obtained. In-depth interviews were conducted either in-person, in a private space within the participants’ workplaces, or by telephone to accommodate their location and privacy preferences. Interviews followed a semi-structured in-depth interview (IDI) guide oriented by the social-ecological model^16^ to explicitly understand individual, household, and community/facility influences on health behaviors and outcomes (Appendix 1) using a flexible approach using open-ended questions and probing techniques to obtain a nuanced understanding of interviewee perspectives and experiences. Interviews lasted between one and two hours and were audio-recorded with participant permission.^17^ Recordings were translated (if not in English) and transcribed for analysis.

### Data analysis

We analyzed the transcripts using both deductive codes, derived from the interview guide and prior literature, and inductive codes, which emerged during transcript review. Five research team members—two Ugandan qualitative researchers (HN and US), an American social epidemiologist (AE), an American qualitative researcher (MG), and an Indian psychiatrist and public health student (SK)—collaboratively developed the coding framework and coded the transcripts in Dedoose software. The team iteratively reviewed jointly coded segments for agreement and resolved coding disagreements through discussion, refining the coding framework as needed. Subsequently, team members individually coded transcripts, discussing any concerns as a group. Individual team members then developed emergent themes within counseling domains, which the full team discussed and interpreted. Data analysis for this manuscript focused on describing counseling practices across the key domains of resuming sexual activity, family planning use, post-repair pregnancy, and post-repair birth. We explored these practices, their variation by patient characteristics, strategies used to support patient adherence to guidelines, and suggestions for improving post-repair counseling.

### Ethical approval

This study was conducted in accordance with the Declaration of Helsinki.Study procedures were reviewed and approved by the University of California, San Francisco Institutional Review Board (IRB# 19–27901), the Mulago Hospital Research and Ethics Committee (MHREC# 1674), and Uganda National Council for Science and Technology (HS #2706). All participants provided written confirmation of informed consent.

## Results

### Participant characteristics

The study included 30 diverse fistula care providers practicing across Uganda: five participants were fistula surgeons, 17 were nurses, 6 were midwives, and two were social workers (Table 1). Most were female (26/30). Median age was 38 years old. Provider experience with fistula care varied, with a few having less than one year and the majority over 5 years of experience. Nine participants were employed at a regional referral hospital, seven at a private not-for-profit hospital, seven at a national referral hospital, four at a mission hospital, and one at a health center. Participants worked across multiple regions of Uganda.

### Provider counseling practices

We present the diverse general counseling practices and specific strategies providers employed across key domains—resumption of sexual activity, family planning, post-repair pregnancy, and post-repair birth—to guide fistula patients and optimize their post-repair outcomes.

#### Resuming sexual activity

Providers universally recommended a 3–6-month delay in resuming sexual intercourse post-repair for healing. While three months was standard, some strategically reported advising patients to wait longer. As one nurse explained, advising six months ensured that patients would observe the minimum three-month period, acknowledging potential early resumption. This “buffer” strategy reveals a pragmatic approach to promoting adherence based on provider experience:

“[…] We always counsel [patients] that after repair they need to not resume sexual activity until after three months in order to have proper healing. Practically, we told them to wait six months because if you tell them to wait three months, they will get involved in sexual activity before the end of three months. We tell them six months so that they wait at least three months and then they will resume sexual activity.”

Nurse, Eastern region

Some providers reported recommending longer delays in resuming sexual activity for patients with more severe fistulas, ongoing post-repair issues, or a history of multiple repair surgeries, indicating a tailored approach based on individual clinical complexity:

“We give them different information because fistulas are of different locations. There are some very bad ones, and in this case, we tell them to delay beyond that (6 months) … There are clients who are too risky because they leave the hospital when they are still leaking and with issues [we tell those] maybe a year.”

Nurse, Eastern region

[…] For those with many repairs […] we will give them a longer period because we are worried about another fistula occurring. It also depends on how you assess the mother, if she’s not okay then we can prolong the period.”

Nurse, Western region

Other providers’ appraisal of the woman’s social circumstances also informed the waiting period advised, as shared below:

[…] There are some who are hard to manage and when you notice such a woman, you advise them to wait 6 months; you sometimes notice a difficult client by just listening to what they say among other patients, and we give them a longer period of time to make sure that she is able to wait at least 3 months.”

Nurse, Eastern region

#### Family planning and contraceptive use

Contraceptive counseling post-fistula repair varied by facility affiliation. Government hospitals offered comprehensive access to modern contraceptive methods. In contrast, religiously-affiliated hospitals, including Catholic missions, primarily focused on natural family planning (fertility awareness or rhythm methods), referring patients elsewhere for modern contraceptives. This divergence, rooted in institutional beliefs, impacted women’s access to their preferred contraceptive methods and posed a barrier to effectively delaying post-repair pregnancy. One provider described:

“Now that I’m working with a catholic-funded setup where family planning is against their beliefs […] we encourage our mothers to use natural family planning methods. For those who prefer artificial methods, it is up to them to choose where to seek family planning services. For those who choose natural family planning, we have a special person for that and even during fistula repair camps, she is there to educate them.”

Fistula surgeon, Eastern region

Providers also discussed individually tailoring contraceptive counseling based on patient age, number of children, partnership status, and fistula type/severity. A key strategy was age and parity-based tailoring: providers predominantly offered long-acting or permanent contraception to older women with higher parity who indicated no desire for future pregnancies while younger women with fewer or no children were primarily counseled on short-term methods to temporarily delay conception.

“Elderly mothers are usually given the long-term methods because surely there is no reason as to why you take an injection, yet you already have so many children like 7 or 8 […] we recommend a copper-T IUD, or a hysterectomy […] for a young woman, it’s basically injections.”

Nurse, Eastern region

“It depends on the characteristic of their fistula and their age bracket. Because they are young adults, the counselling I give to the 40-year-old women, it’s different from the one that I give to the 15 or 16-year-olds.

Nurse, Central region

Providers also emphasized fistula characteristics (type, severity, continence) and obstetric history (multiple repairs, recurrence) as key contraceptive counseling factors. Providers prioritized risk mitigation, recommending longer-acting methods for women with more complex fistula histories or those at higher risk of recurrence. Counseling for repeat fistula cases focused on addressing potential causes (sexual activity/conception and delivery mode) to prevent future occurrences.

“It depends on the age bracket and the type of fistula. Even those that have had RVF (recto vaginal fistula), their counseling is quite different also. The greatest emphasis is on family planning for those getting RVF for the second time […] we’ll emphasize what might have caused that fistula to reoccur”

Nurse, Central region

“[…] those who had more than two surgeries need to use family planning for a longer period of time, and they also opt for family planning methods that will last a long time.”

Nurse, Western region

#### Post-repair pregnancy

All providers emphasized the importance of delaying pregnancy for proper healing, yet the recommended timelines varied widely (3 months to 2+ years), even within facilities, attributed to the lack of standard counseling guidelines. Despite this discordance, providers described individually tailoring guidance based on patient factors: longer delays were advised for women with severe or complex fistulas, multiple repairs, poor obstetric history, or high parity, while shorter delays were suggested for younger women with fewer children.

“[…] doctor always tells them to wait 6 months to 1 year depending on the severity and type of fistula that they had […] those who had big fistulas and underwent several repairs wait a bit longer […] more than a year and it is mothers who had minor fistulas who wait 6 months.”

Nurse, Western region

“There are also those with complicated fistulas and they have had extensive uterine repair […] you could even ask them to wait for at least 2–3 years because you will realize that if she conceives quickly, she might end up getting serious damage. But for the minors, we usually tell them to take at least one year. With RVF (recto-vaginal fistula) we usually tell them to take one year and a half [years]”

Nurse, Eastern region

#### Post-repair childbirth

Nearly all providers recommended elective cesarean surgery for all births following fistula repair, regardless of fistula characteristics or obstetric history. This guidance prioritized preventing fistula recurrence associated with vaginal birth. Providers emphasized planning for cesarean surgery before labor onset and early in the pregnancy, consistent antenatal care with disclosure of fistula history to facilitate this plan, and close monitoring throughout pregnancy to minimize recurrence risk.

“We tell them to give birth via cesarean section and not to have a normal delivery because it’s during normal delivery that they get complications. We also tell them not to wait for labor. They’re not supposed to get any labor pains because when a woman starts labor, the child starts descending looking for a path to come out […] we advise her to come at 7 months to do a scan to confirm the date of operation and when their child will be ready for delivery so, we will give her an elective surgery before she goes into labor.”

Nurse, Central region

“However, we advise them, ‘once you get pregnant, go and start antenatal care and please go with your card, wherever you are going to seek antenatal care,” we tell them to present the cards to the doctor and tell them about the repair so that they can plan for CS since they’re not allowed to labor or push…”

Nurse, Western region

A few providers suggested that vaginal birth might not always elevate risk post-repair, particularly for less complex fistulas. However, they expressed concern that nuanced messaging could confuse patients and increase risk, given the limited care continuity between fistula and obstetrics providers, supporting the prevailing recommendation for universal elective cesarean.

“Theoretically if she had a high injury following a cesarean section, she may be able to push because it is not related to the pelvis itself […] we tell them that they should deliver by an elective caesarean section once they have had VVF or RVF even if it was iatrogenic […] we don’t want them to try.”

Fistula surgeon, Central region

Providers varied in their reports of recommended presentation timeline for planned cesarean section, typically around 38 weeks, although some providers requested an earlier visit around seven months for later gestational age confirmation. This inconsistency catered to differing expectations about patients’ awareness of their true gestational age, potentially impacting timely access to care.

“At 38 weeks they have to go to the hospital and wait there because even when I am not at the ward, they’re given priority because they are risky mothers.”

Nurse, Western region

Providers offered varied advice on where to seek post-repair maternity care. Some recommended accessing higher-level facilities in general (e.g., a general hospital or higher for cesarean availability) and emphasized disclosure of fistula history to childbirth providers.

“We tell them to go to a general hospital when they get pregnant and to only go to a small clinic for minor checkups. They are supposed to give birth from a general [hospital] and shouldn’t be lied to that she will be able to push. She needs to make sure that she discloses to the doctor or midwife that she had a fistula and that after repair, she was advised to have a C-section.… Some of them [follow this advice]; however, some go back to small health centres where they get complications again”

Midwife, Central region

Other providers specifically advocated returning to the fistula repair facility for continuity of care, with some facilities even offering early booking and financial assistance for cesarean surgery.

“We don’t allow these mothers to give birth in rural areas or to go into labor.… At 7 months they have to come here for a scan so that we can book them for the C-section. We do this to prevent the same problem and also prevent any damage to the repair site. We pay for the [surgery] expenses … as a way of preventing repeat fistulas and give them the joy of having a child.”

Nurse, Central region

### Provider strategies for effective counseling

Providers across all four main counseling domains (i.e., resumption of sexual activity, family planning, post-repair pregnancy, and post-repair childbirth) employed diverse strategies centered on partner and household engagement, temporary partner separation when needed, peer support, and provision of written discharge instructions in local languages. Further, ensuring care continuity and comprehensive counseling services, including addressing infertility and economic empowerment, were considered important for improving post-repair outcomes.

#### Partner and household engagement

Engaging male partners in counseling, both at the facility and in the community, was a key cross-cutting strategy to improve post-repair guideline adherence. Providers identified some male partners as barriers to women meeting post-repair guidelines and found that involving them directly helped secure their understanding and support, particularly for observing the recommended waiting period for resuming sexual activity:

“[the women] will always come back and tell you, ‘I thought that I should wait however my husband wants me to engage in sexual intercourse. What should I do?’ […] actually, one of these men told me that he thought that it was the wife making up these things. We need to have both parties around so that the gentlemen understand why the woman has to wait and that he has to be patient so that this mother can recover.”

Social worker, Central region

Providers from multiple facilities reported couples counseling as a strategy to educate male partners on the purpose of the delay and to help them empathize with their female partners’ condition.

“[…] When their partners are educated, physically or through a phone call, they are very supportive and follow the guidelines. […] we do the [male partner counselling] here at the hospital. They are at times ready to listen and understand their input, especially the married ones.”

Social worker, Central region

Providers also welcomed or invited partners to counseling sessions to educate them of the importance of delaying pregnancy for the woman’s recovery and to enlist their support in adhering to the guidelines, often emphasizing family planning use.

“We ask [women] to give me their spouses’ contacts so that we can explain it to them. Sometimes we ask them to bring their partners to the facility. We ask them to delay pregnancy from occurring one year after repair and in this case, we’ll advise them to family planning methods like condoms.”

Nurse, Northern region

#### Temporary partner separation

Recognizing potential objections from male partners, some providers advised temporary separation for women concerned about adhering to the recommended delays for resuming sexual activity and post-repair pregnancy. This included suggesting stays with relatives, separate sleeping arrangements within the home, or a return to boarding school for younger in-school patients.

“[…] We also advise them to go live with a relative where the man won’t be because sometimes these men don’t really understand the importance of waiting three months.”

Social worker, Central region

“[…]the advice which we gave is that they can sleep in separate rooms but can share a meal on the dining table but not share bedrooms […] so that her husband can understand the essence of waiting.”

Social worker, Central region

Facilities also utilized post-repair reintegration programs, including temporary housing and economic and social empowerment activities for 2–3 months, as a strategy to indirectly facilitate partner separation and support adherence to recommended waiting times.

“[…] This program trains them on tailoring, weaving, and hairstyling. They stay there […] this has helped us a lot because, by the time they go back home, the [sexual resumption] waiting period would have passed.”

Nurse, Eastern region

“[…] We take on a full integration, so that she can delay getting close to a man, by training them in income-generating activities like tailoring and explaining to them the dangers of getting involved in sex so early after repair.”

Midwife, Eastern region

#### Peer counseling

One facility described using peer counselors, women who successfully became pregnant and had healthy children after having had a fistula repair, to counsel other women and their partners. These individuals shared their successful navigation of post-repair pregnancy following provider recommendations.

“We also told them that there are other women who have had their repairs done. Good enough some of those women are usually at the ward for reviews from the previous camp. So, these new patients get to see their fellow patients that have healed, and they are encouraged that they can get better.”

Administrator, Central region

“We receive men who come to take care of their wives, and they get to know what these women go through, so we also advise them to sensitize fellow men about caring for their wives when they are pregnant.”

Nurse, Central region

#### Written discharge instructions

Some providers utilized written discharge instructions, provided in accessible languages, to reinforce verbal counseling on post-repair recovery, especially for sexual activity resumption guidance. In some facilities this complemented couples counseling to increase male partner support.

“[…] We give them a printed copy of the instructions. When they arrive home, and the woman is interested in sexual intercourse, the husband will tell her, ‘We were told to wait 3 months’ […] We write them down in Lusoga or English and they each choose a language that they can read.”

Nurse, Eastern region

Providers at four facilities reported giving patients a discharge card containing key information: educational reminders, important dates, and fistula diagnosis details. This card was intended to facilitate communication with future antenatal care providers and ensure appropriate management, including planned cesarean.

“… we give them a discharge card that includes information about these mothers. We explain what we have done for them and what we have told her to do. For example, not having normal labor. We also include issues to do with resuming sexual intercourse. So, when they’re going for antenatal care, we advise them to go with that card and present it to our health provider so that they are aware that about their complication.”

Nurse, Central region

“We provide them with a written document that they present at the hospital that they go to so that the c-section can be done for them since they have proof that they had fistula repair.”

Nurse & trainer, Eastern region

#### Care continuity

Providers emphasized the importance of care continuity by either providing contraceptive methods or linking women to family planning clinics. Due to the differing timelines regarding the safety of resuming sexual intercourse post-repair and becoming pregnant, comprehensive contraceptive counseling was seen as vital for women to choose preferred methods. While most referred patients, some facilities provided methods directly, addressing access challenges post-discharge.

“We have family planning clinics that are specialized, so we don’t go into details about it. However, we refer them to the clinics after 6 weeks. When they come back for review, we tell them, ‘It is best to go to the family planning clinic and talk to the health workers there so that you are initiated into it.’”

Midwife, Central region

“So, usually, we give them the implant and some of them take pills depending on what they prefer. Within the care centers, I think we should be able to give family planning methods to these mothers as they go out so that it is catered for, otherwise, if they go out, family planning is the last thing on their mind until it is too late.”

Fistula surgeon, Central region

To enhance care continuity, providers actively facilitated patients’ access to contraception before discharge, sometimes through direct escort to on-site family planning clinics. Additionally, some facilities implemented post-discharge follow-up systems (i.e., phone calls, informal check-ins) to provide ongoing support, address challenges, and offer further education and referrals.

“I make a phone call to the in-charge to inform her about these patients, and I also followed up to know whether she received them. Sometimes when I’m not busy I walk them to the clinic myself and if I have students par around, they help me do that.”

Social worker, Central region

Providers also described sharing their personal phone numbers with their patients and requesting them to call if they become pregnant for information on where to seek care and how to navigate post-repair pregnancy care needs. Some providers also directly communicated with patients’ antenatal and childbirth care providers to ensure proper information sharing.

“They call or we call them using the phone numbers that we share with them, which makes communication easier because we get to recommend professionals to them. The phone numbers we share with them have made follow-up so helpful. We told them to immediately come to us so that they could get a diagnosis early. [The patients] share their personal phone numbers with health workers.”

Social worker, Central region

Providers emphasized the importance of early and consistent antenatal care, directing women to facilities capable of cesarean section and where disclosure of fistula history and request for elective cesarean would be taken seriously. They also encouraged early planning for elective cesarean birth upon pregnancy confirmation.

They should be encouraged to go for antenatal care so that they can interact with their service providers, so that they get to understand what they went through and prepare a proper service delivery. […] we give them all the antenatal care services and treat all the issues that they have, for example, hypertension and diabetes. They shouldn’t wait for labor so when they are due, they should just go to the facility and wait there.”

Midwife, Eastern region

#### Comprehensive counseling services

Across counseling domains, providers recommended more comprehensive post-repair counseling, emphasizing the necessity of time and training to build strong patient connections for sensitive discussions and ensure the delivery of accurate information.

“The way a health worker counsels these mothers matters a lot. If the health worker attending to the mother has less information about VVF, then they may not give adequate information to this client or may give the wrong information.”

Nurse, Central region

A critical challenge is the limited number and availability of knowledgeable fistula care providers, which can lead to patient discouragement, repeated unsuccessful attempts to seek care, and potentially the adoption of risky alternatives due to unmet needs.

“I think we have few numbers of providers […] [when patients] come to the hospital for help, and the providers who are supposed to support them are not there, of course they go back without getting what they had come for. If they move to the hospital twice or thrice without getting this provider, they will give up and say, ‘let me take this risk and do it like this.’”

Nurse, Northern region

Providers highlighted the importance of effective counseling to proactively motivate patients to follow recovery guidelines. They emphasized the negative consequences of too-early sexual activity or pregnancy and reminded patients of their past physical and psychological trauma as a means of gaining compliance.

“[…] You tell them, ‘It’s you who was admitted here and you were the one leaking urine. Not anyone else! So, it’s your life and you need to make sure that this repair heals before you resume sex.’”

Nurse, Eastern region

“… when she is aware that if she gets pregnant, she would get the problem again, she will try so hard to follow the guidance you gave her. She would go and do what she has to do. We usually inform them about how these hormones work because if you teach her on how it works, she knows that if I use this, this is how it works to prevent pregnancy.…. When they understand that information, they can use it very well”

Midwife, Central region

Providers also underscored the importance of sharing thorough and comprehensive information to enable patients to make informed decisions regarding the risks of post-repair pregnancy. They sought to include all the varied considerations while respecting the patient’s decision-making autonomy.

“We provide [patients] enough information to help them achieve this guidance and it has been so good so far because they at times involve us in their decisions. We also let them know that “you’re not the only ones that have been here and if you take in the information the midwives and doctors give, you will get better lives.’”

Social worker, Central region

They also shared the importance of education on post-repair pregnancy and family planning, explaining contraception to support informed decisions and consistent use, as well as ensuring access to family planning methods prior to discharge.

“We encourage them to make sure that they go back home with their family planning in place already. We counsel them and they should understand the dangers of conceiving early so that they maintain their family planning and keep on getting the dose”

Midwife, Central region

Lastly, providers emphasized the need for sensitivity in counseling to address post-repair infertility, particularly after women who had previously undergone hysterectomy due to obstetric complications (e.g., ruptured uterus, etc.). This involved explaining uterine function and the impossibility of future conception, while also clearly explaining the emergency circumstances necessitating the surgery to support and promote acceptance.

“I tell [women]: ‘you had an emergency while giving birth and your uterus ruptured beyond repair or was damaged and had to be removed to save your life. With this, you won’t be able to conceive or have your periods.’”

Fistula surgeon, Eastern region

Providers also emphasized assessing and addressing social and familial support and offering counseling where support is lacking.

“…We also talk to those they are staying with[…] we also ask if they are getting people that are misleading them like sisters-in-law. When they tell you ‘yes’, we also bring that person on board. If she says that she doesn’t want to fetch water or carry heavy work, we bring them on board and explain it to them although it’s not easy.”

Midwife, Central region

#### Economic empowerment

Providers shared that post-repair counseling should prioritize economic empowerment, including counseling women around economic self-sufficiency and referring to vocational training programs to reduce risks due to socioeconomic vulnerability. Providers felt providing these services helped mitigate early sexual resumption and post-repair pregnancy.

“We love to empower these women. Poverty is also a problem, so we told them to find something to do instead of waiting for their men to provide money to take care of their homes. […] we had funds that we would give to these women after they were trained to do some things. We used to call that a “start kick”, this would give these women some start up-[…] could maybe rear goats, pigs, or chickens, tailoring or hair styling.”

Administrator, Central region

“We advise women to engage in other activities and that is when the NGOs come in to rehabilitate them. If you have something you are doing, because as we have seen, whatever led them into pregnancy was [lack of] money. So, if you are trained to plait hair and you can earn a little money, it would reduce the risk.”

Theatre assistant nurse, Central region

### Key challenges

Participant-perceived challenges to effective post-repair pregnancy counseling were largely systemic. These include a lack of standardized guidelines and insufficient provider expertise, leading to inconsistent and potentially inadequate patient support.

“There’s no standardization because … there are a lot of unknowns and people don’t use science but dogma”

Fistula surgeon, Eastern region

“Some of us lack knowledge about these things, and [some providers] don’t know that these women are supposed to go with the contraceptive after discharge, and the clients themselves tend to miss or take it as an opportunity”

Nurse, Northern region

Providers described other challenges including limited follow-up care, marked by poor access to family planning and reintegration services, along with fragmented care coordination and financial constraints, lack of supplies (e.g., contraceptive stock-outs), and the costs of guideline-specific care (e.g. elective cesarean section). Providers shared the following comments as they discussed these diverse challenges related to counselling around contraceptives and post-repair childbirth-related care:

“We offer counselling about the methods, and they even leave after making a choice, however, we don’t offer these services here. So, if it is not offered at a facility where they are handled, this will hinder them from getting the service because they have to move from one facility to another. We lose patients along the way.”

Midwife, Eastern region

In a normal functioning healthcare system, [the obstetric team (for the post-repair pregnancy) and the surgical team who repaired the fistula should come together and decide the best care for these patients] is how it should be but as I say, “there are many gaps in our healthcare system.” So, sometimes they don’t. At times, the patients don’t carry the [VVF discharge] cards … so during antenatal care, this information must be captured by the midwives or anybody offering antenatal care services to these clients.”

Fistula surgeon, Eastern region

As discussions shifted from challenges to potential solutions, participants’ recommendations for improving post-repair counseling effectiveness included establishing clear post-repair counselling protocols, provider training to improve knowledge and skills across key counseling topics, including solution-oriented counseling, and facilitation of subsequent care needs. For example, one provider suggested that tangible problem-solving is needed to overcome barriers to guideline implementation:

“[What we do wrong is to] just give them the information by telling them, “You must deliver by an elective caesarean section. After abstaining, they delay pregnancy and then deliver by an elective C-section. I believe that we just assume that they will just take our word when we say “deliver this way” because we don’t follow them up…. We’re giving them the information and assume that they will take it the way we give it to them, but when they come back with reoccurrences, we don’t know where to put the blame because we don’t want to blame ourselves or the other health workers.”

Fistula surgeon, Central region

Another provider shared that the cesarean section that women with history of fistula should undergo in a post-repair birth should be free of cost to the patients, but also reflected on the sustainability concerns for these services:

“After, we pay for the expenses of the C-section as a way of preventing repeat fistulas and also give them the joy of having a child. [Payment for the C-section] was started by the first doctors who came from abroad; they got funds to cover the C-sections for our repaired mothers and as we speak, we have over 500 children delivered by C-section.”

Nurse, Central region

## Discussion

Our study examined the counseling messages and strategies employed by fistula care providers in Uganda to guide patients across four key domains after female genital fistula repair: resumption of sexual activity, family planning, post-repair pregnancy, and post-repair childbirth. Provider narratives unveiled largely similar counseling content yet identified significant areas of divergence, highlighting the absence of standardized guidelines. Barriers to providing effective post-repair counseling were identified at the individual and systems level across all domains, leading to key recommendations for improvement. Overall, these findings highlight the critical importance of effective provider counseling in optimizing post-repair outcomes for fistula patients and identify systems level opportunities for supporting this goal.

Provider narratives describe an environment in which provider experience and practical modifications are consistently required to compensate for the absence of standardized guidelines.^22^ Substantial inconsistencies in counseling on the recommended time to delay resumption of sexual activity and in particular, time to delay post-repair pregnancy, were identified across providers including among providers of the same cadre and working in the same facility. The large inconsistencies in timing to pregnancy, characterized by the wide range of suggested delays, i.e., 3 months to greater than 2 years, highlights the need for clear, uniform, and evidence-based guidelines for post-repair counseling which are largely missing.^19,23^

Despite these inconsistencies, our study also revealed specific approaches employed by health care providers and facilities to support women through the post-repair period. Providers often tailored advice based on individual patient characteristics including age and parity, as evidenced by the differing recommendations for contraceptive methods: longer-acting or permanent options were discussed ed for older, high-parity women, while methods such as injections were discussed for younger women. Optimally, patients would have a range of choices to make an informed decision.^24^ In some of the scenarios shared by providers, this practice was concordant with patient fertility preferences, reflecting providers’ voiced considerations of the importance of patient-centered counseling and in alignment with setting-specific social norms and prevalent fistula-related stigma.^25^ In other scenarios, as reflected in provider narratives of some higher parity women, provider-patient discordance around the importance of subsequent childbearing versus the risk was evident. Systemic challenges, including stock-outs of preferred contraceptive methods and geographical barriers to accessing family planning clinics, impeded women’s ability to take up these recommendations, concurrent with previous findings.^26–28^ The influence of facility level characteristics was also pronounced, with religiously affiliated hospitals primarily promoting natural family planning, thereby limiting the range of contraceptive options and potentially impacting patient autonomy and choice.^29,30^

Providers shared their use of pragmatic approaches to promoting adherence to sexual resumption guidelines, including advising longer waiting periods as a “*buffer*” (e.g., suggesting six months to ensure at least three months of abstinence), engaging male partners in counseling, leveraging peer support, and providing written guidance in locally accessible languages. Yet, these strategies were often undermined by patient-related challenges such as low literacy, low socioeconomic status, and limited agency due to socioeconomic vulnerabilities. Similar research documents these challenges among fistula survivors as a major factor in non-adherence to health provider recommendations and especially in the post-repair recovery period.^31^ Providers also underscored the importance of male involvement in counseling, especially as it relates to resumption of sexual activity, emphasizing the need for inclusive, culturally sensitive and context-specific counseling approaches as has been identified as important for optimizing other reproductive and sexual health behaviors.^32^

A key and largely unaddressed major challenge experienced by providers was the limited follow-up of fistula care and the lack of strong care networks linking fistula care to other reproductive and perinatal health care. In context of fragmented health care systems, providers attributed the lack of continued support and care continuity to the absence of an integrated system for supporting fistula survivors, as shown in previous studies.^22,33^ This largely influenced the counseling effectiveness of post-repair pregnancy delay and birth mode for post-repair pregnancies. For example, providers in our study expressed strong consensus regarding the importance of elective cesarean section for post-repair births to prevent recurrence,^14^ with most providers reporting vaginal childbirth following fistula repair as a primary contributor to fistula recurrence. However, in environment where care networks are generally not well connected, a domain frequently cited as a challenge within the Ugandan healthcare system,^34^ significant provider burden to employ diverse strategies in counseling around these issues to increase adherence was evident.^35^ Strategies that were mentioned ranging from recommending accessing specific childbirth facilities, early engagement in antenatal care, tools to facilitate fistula history disclosure, early presentation to childbirth care, and staying connected to the fistula care provider for guidance. Prevalent misinformation, individual preferences and social norms about cesarean birth, and socioeconomic vulnerability provided further challenges to the effectiveness of this specific counseling domain.^36–38^ Other challenges included the lack of fistula knowledge among some perinatal care providers, highlighting the need for education for improving awareness of best practices for fistula among all healthcare providers.^34^

Despite these challenges, providers in our study demonstrated creativity and resourcefulness in the development and implementation of strategies to enhance counseling effectiveness for improve fistula repair outcomes, highlighting their personal dedication to their patients. The range of strategies from engaging support persons (e.g., male partners, other family members, other women with fistula), providing written instructions and documentation for providers, and actively facilitating care continuity through referrals and even direct accompaniment to family planning clinics showcased their nuanced appreciation of the multi-level barriers and facilitators for optimizing fistula repair outcomes. Fistula provider cultural competence was also emphasized in their prioritization of sensitively addressing challenging topics, reflecting best practices.^39^ For example, they described addressing post-repair infertility concerns with compassion, including carefully explaining the necessity of prior hysterectomies while acknowledging the loss of future childbearing. Practices and recommendations with broad potential direct and indirect impacts also included the incorporation of economic empowerment initiatives, such as formal fistula reintegration programs and referrals to vocational training programs.^19,33,40,41^ The provision of “start-up” funds or resources for income-generating activities was seen to mitigate socioeconomic vulnerabilities that could lead to early resumption of sexual activity or unintended pregnancies and has previously shown success.^33,41,42^

### Strengths and limitations

Our study contributes to the literature on practices and challenges around post-repair counseling for fistula survivors in Uganda, documenting the perspectives and experiences of 30 diverse providers from varied facility types, regions, and professional backgrounds (i.e., nurses, surgeons, counselors, social workers, and administrators). Our approach highlights the context-specific challenges, and strategies employed by providers in the absence of standardized guidelines for post-repair counseling. The cadre range covered in our study incorporates ensures that the voices of all along the fistula care continuum of care are included, adding depth to the data and enhancing the applicability of findings within the Ugandan context. Secondly, our data analysis approach, a team-based collaborative process in which the data collectors are also involved in the analysis and interpretation, renders the findings more robust. However, several limitations need to be mentioned. The sample size of 30 providers, although common in qualitative research, may not be representative of all fistula care providers in Uganda, limiting the generalizability of the findings. As with all qualitative research relying on interview data, the potential for researcher bias in the interpretation of provider reports and recall bias in their descriptions of past practices cannot be discounted, but we note the participation of numerous team members in coding and interpretation, enhancing the rigor and validity of results. In addition, the focus of the study on providers’ perceptions alone provides critical documentation of experiences and perceived obstacles but not of fistula survivors themselves. The inclusion of patient narratives in future research will provide a more complete and triangulated understanding of current counseling practices and their effectiveness.

## Conclusion

This study highlights the critical importance of effective provider counseling in optimizing post-repair outcomes for fistula patients. By employing diverse strategies and tailoring guidance to individual patient needs, providers can significantly improve recovery and long-term health outcomes. Developing and implementing clear guidelines, addressing systemic challenges, and enhancing provider training and care continuity are essential for improving post-repair counseling effectiveness and ensuring comprehensive support for fistula patients.

## Supplementary Material

Supplementary Files

This is a list of supplementary files associated with this preprint. Click to download.

- Appendix1ProviderIDIGuide.docx

## Figures and Tables

**Table 1.** Characteristics of Fistula Care Provider Study Participants (N=30)

	n	%
Age (median, iqr)	38 (32–52)	
Gender		
Female	26	87
Male	4	13
Cadre		
Surgeon	5	17
Nurse	17	57
Midwife	6	20
Social worker	2	7
Time in fistula care		
<1y	3	10
1–2y	1	3
3–5y	9	30
6–10y	8	27
>10y	9	30
Patients seen per year (median, iqr)	55 (30–67)	
Involved in provider training		
Yes	8	27
No	22	73
Facility type		
Regional referral hospital (n=4)	9	30
Private not-for-profit hospital (n=1)	7	23
National referral hospital (n=1)	7	23
Mission hospital (n=4)	4	13
Health center IV (n=1)	1	3
District		
Central	12	40
Eastern	13	43
Northern	2	7
Western	3	10

## Data Availability

Data are available at reasonable request from the corresponding author.

## References

[1] UN. General Assembly (77th sess.: 2022–2023), editor. Intensification of efforts to end obstetric fistula: resolution. New York: UN, 30 https://digitallibrary.un.org/record/3999353 (accessed July 29, 2025).

[2] Hilton P. Vesico-vaginal fistulas in developing countries. Int J Gynaecol Obstet. 2003;82:285–95. 14499975 10.1016/s0020-7292(03)00222-4

[3] Hilton P, Ward A. Epidemiological and surgical aspects of urogenital fistulae: a review of 25 years’ experience in southeast Nigeria. Int Urogynecol J Pelvic Floor Dysfunct. 1998;9:189–94. 9795822 10.1007/BF01901602

[4] Arrowsmith S, Hamlin EC, Wall LL. Obstructed labor injury complex: obstetric fistula formation and the multifaceted morbidity of maternal birth trauma in the developing world. Obstet Gynecol Surv. 1996;51:568–74. 8873157 10.1097/00006254-199609000-00024

[5] Wall LL, Arrowsmith SD, Briggs ND, Lassey A. Urinary Incontinence in the Developing World: The Obstetric Fistula. In: Abrams P, Cardozo L, Khoury S, Wein A, editors. Incontinence. Plymouth, U.K.: Health Publication Ltd; 2002.

[6] Wall LL, Arrowsmith SD, Briggs ND, Browning A, Lassey A. The obstetric vesicovaginal fistula in the developing world. Obstet Gynecol Surv. 2005;60:S3–51. 16034313 10.1097/00006254-200507001-00002

[7] Zeleke BM, Ayele TA, Woldetsadik MA, Bisetegn TA, Adane AA. Depression among women with obstetric fistula, and pelvic organ prolapse in northwest Ethiopia. BMC Psychiatry. 2013;13:236. 24070342 10.1186/1471-244X-13-236 PMC3849390

[8] Browning A, Fentahun W, Goh JT. The impact of surgical treatment on the mental health of women with obstetric fistula. BJOG. 2007;114:1439–41. 17903234 10.1111/j.1471-0528.2007.01419.x

[9] Goh JT, Sloane KM, Krause HG, Browning A, Akhter S. Mental health screening in women with genital tract fistulae. BJOG. 2005;112:1328–30. 16101616 10.1111/j.1471-0528.2005.00712.x

[10] Wilson AL, Chipeta E, Kalilani-Phiri L, Taulo F, Tsui AO. Fertility and pregnancy outcomes among women with obstetric fistula in rural Malawi. Int J Gynaecol Obstet. 2011;113:196–8. 21457981 10.1016/j.ijgo.2011.01.006

[11] Kopp DM, Tang JH, Bengtson AM, Continence, quality of life, and depression following surgical repair of obstetric vesicovaginal fistula: a cohort study. BJOG Int J Obstet Gynaecol. 2019;126:926–34. 10.1111/1471-0528.15546 PMC6510632 30461170

[12] Delamou A, Utz B, Delvaux T, Pregnancy and childbirth after repair of obstetric fistula in sub-Saharan Africa: Scoping Review. Trop Med Int Health. 2016;21:1348–65. 27596732 10.1111/tmi.12771

[13] Drew LB, Wilkinson JP, Nundwe W, Long-term outcomes for women after obstetric fistula repair in Lilongwe, Malawi: a qualitative study. BMC Pregnancy Childbirth. 2016;16:2. 26732574 10.1186/s12884-015-0755-1 PMC4702356

[14] Korn AP, Barageine JK, Nalubwama H, Pregnancy outcomes following surgical repair of female genital fistula in Uganda. AJOG Glob Rep. 2025;5:100481. 40264448 10.1016/j.xagr.2025.100481 PMC12013486

[15] Browning A. Pregnancy following obstetric fistula repair, the management of delivery. BJOG Int J Obstet Gynaecol. 2009;116:1265–7. 10.1111/j.1471-0528.2009.02182.x 19438494

[16] de Bernis L. Obstetric fistula: guiding principles for clinical management and programme development, a new WHO guideline. Int J Gynaecol Obstet. 2007;99(Suppl 1):S117–21. 17880979 10.1016/j.ijgo.2007.06.032

[17] Engender Health. Family Planning for Women and Couples following Fistula Repair. New, York NY. 2010 https://www.engenderhealth.org/wp-content/uploads/2021/10/Family-Planning-for-women-and-couples-following-Fistula-repair.pdf

[18] Browning A. Risk factors for developing residual urinary incontinence after obstetric fistula repair. BJOG. 2006;113:482–5. 16489933 10.1111/j.1471-0528.2006.00875.x

[19] United Nations. Obstetric Fistula & Other Forms Of Female Genital Fistula. U. N. Popul. Fund. https://www.unfpa.org/publications/obstetric-fistula-other-forms-female-genital-fistula (accessed Nov 20, 2023).

[20] Uganda Bureau of Statistics. Uganda Demographic and Health Survey 2022: Main Report. Kampala, Uganda: Uganda Bureau of Statistics and ICF; 2023.

[21] Maheu-Giroux M, Filippi V, Samadoulougou S, Prevalence of symptoms of vaginal fistula in 19 sub-Saharan Africa countries: a meta-analysis of national household survey data. Lancet Glob Health. 2015;3:e271–8. 25889469 10.1016/S2214-109X(14)70348-1

[22] Ruder B, Emasu A. The Promise and Neglect of Follow-up Care in Obstetric Fistula Treatment in Uganda. In: Wallace LJ, MacDonald ME, Storeng KT, eds. Anthropologies of Global Maternal and Reproductive Health: From Policy Spaces to Sites of Practice. Cham (CH): Springer, 2022. http://www.ncbi.nlm.nih.gov/books/NBK584053/ (accessed July 29, 2025). 36108110

[23] Fistula Care Plus Project. Counseling the Obstetric Fistula Client. 2012 https://fistulacare.org/archive/files/3/3.1/counseling-curriculum-english.pdf

[24] World Health Organization. Family Planning: A Global Handbook for Providers. Geneva, Switzerland: World Health Organization; 2022.

[25] Atahigwa C, Coene G, Achen D, Neema Murembe C, Lafaut D. Navigating Gender and Power: A Qualitative Analysis of Fertility Decision-Making in Rubirizi District, Southwestern Uganda. Womens Reprod Health; 0: 1–13.

[26] Kristiansen D, Boyle EH, Svec J. The impact of local supply of popular contraceptives on women’s use of family planning: findings from performance-monitoring-for-action in seven sub-Saharan African countries. Reprod Health. 2023;20:171. 37990268 10.1186/s12978-023-01708-7 PMC10664543

[27] Muhoza P, Koffi AK, Anglewicz P, Modern contraceptive availability and stockouts: a multi-country analysis of trends in supply and consumption. Health Policy Plan. 2021;36:273–87. 33454786 10.1093/heapol/czaa197 PMC8058948

[28] Yao J, Murray AT, Agadjanian V. A geographical perspective on access to sexual and reproductive health care for women in rural Africa. Soc Sci Med. 2013;96:60–8. 24034952 10.1016/j.socscimed.2013.07.025 PMC4609647

[29] O’Brien J. Can faith and freedom co-exist? When faith-based health providers and women’s needs clash. Gend Dev. 2017;25:37–51.

[30] Tafesse W, Chalkley M. Faith-based provision of sexual and reproductive healthcare in Malawi. Soc Sci Med. 2021;282:113997. 34183195 10.1016/j.socscimed.2021.113997

[31] Tilahun M, Nalubwama H, Getahun M, Barageine JK, El Ayadi AM. Understanding Women’s Pregnancy Intentions, Decision-Making, and Factors Influencing Reproductive Choices After Genital Fistula Repair in Uganda: A Qualitative Study. PLOS Glob Public Health. 2025;5:e0004015. 40215209 10.1371/journal.pgph.0004015 PMC11990634

[32] Aventin Á, Robinson M, Hanratty J, Involving men and boys in family planning: A systematic review of the effective components and characteristics of complex interventions in low- and middle-income countries. Campbell Syst Rev. 2023;19:e1296. 36911859 10.1002/cl2.1296 PMC9837728

[33] El Ayadi AM, Painter CE, Delamou A, Rehabilitation and reintegration programming adjunct to female genital fistula surgery: A systematic scoping review. Int J Gynaecol Obstet Off Organ Int Fed Gynaecol Obstet. 2020;148(Suppl 1):42–58. 10.1002/ijgo.13039 PMC7003948 31943181

[34] Kanyesigye H, Ngonzi J, Mulogo E, Fajardo Y, Kabakyenga J. Health Care Workers’ Experiences, Challenges of Obstetric Referral Processes and Self-Reported Solutions in South Western Uganda: Mixed Methods Study. Risk Manag Healthc Policy. 2022;15:1869–86. 36225611 10.2147/RMHP.S377304 PMC9550169

[35] Kalaris K, Radovich E, Carmone AE, Networks of Care: An Approach to Improving Maternal and Newborn Health. Glob Health Sci Pract. 2022;10:e2200162. 36562444 10.9745/GHSP-D-22-00162 PMC9771468

[36] Harrison MS, Goldenberg RL. Cesarean section in sub-Saharan Africa. Matern Health NeonatolPerinatol. 2016;2:6. 10.1186/s40748-016-0033-x PMC4937522 27398224

[37] Atuheire EB, Opio DN, Kadobera D, Spatial and temporal trends of cesarean deliveries in Uganda: 2012–2016. BMC Pregnancy Childbirth. 2019;19:132. 30991975 10.1186/s12884-019-2279-6 PMC6469217

[38] Dusabe J, Akuze J, Kisakye AN, Kwesiga B, Nsubuga P, Ekirapa E. A case-control study of factors associated with caesarean sections at health facilities in Kabarole District, Western Uganda, 2016. Pan Afr Med J. 2018;29:179. 30050643 10.11604/pamj.2018.29.179.14870 PMC6057596

[39] Johnson KA, Turan JM, Hailemariam L, Mengsteab E, Jena D, Polan ML. The role of counseling for obstetric fistula patients: Lessons learned from Eritrea. Patient Educ Couns. 2010;80:262–5. 20034756 10.1016/j.pec.2009.11.010 PMC3552555

[40] Gondwe MS, Maharaj P, Sewpersad S. The reintegration of obstetric fistula survivors in Malawi: Perspectives of healthcare providers. Midwifery. 2023;126:103834. 37782973 10.1016/j.midw.2023.103834

[41] Ruder B, Emasu A. Making the Case for Holistic Fistula Care: Implementation of a Model Reintegration Program in Uganda. A Multidisciplinary Approach to Obstetric Fistula in Africa. Cham: Springer; 2022. pp. 429–40.

[42] El Ayadi AM, Alway J, Matityahu D, Kichwen C, Wilson S, Mabeya H. Impact of Beyond Fistula programming on economic, psychosocial and empowerment outcomes following female genital fistula repair: A retrospective study. Int J Gynaecol Obstet Off Organ Int Fed Gynaecol Obstet. 2023. 10.1002/ijgo.15133. published online Sept 25. 37746937

